# Effective Techniques for Multimodal Data Fusion: A Comparative Analysis

**DOI:** 10.3390/s23052381

**Published:** 2023-02-21

**Authors:** Maciej Pawłowski, Anna Wróblewska, Sylwia Sysko-Romańczuk

**Affiliations:** 1 Faculty of Mathematics and Information Science, Warsaw University of Technology, Koszykowa Street 75, 00-662 Warsaw, Poland; 2 WeSub, Adama Branickiego Street 17, 02-972 Warsaw, Poland; 3 Faculty of Management, Warsaw University of Technology, Narbutta Street 85, 02-524 Warsaw, Poland

**Keywords:** multimodal representation, multimodal learning, data fusion, deep learning in sensor systems, comparative analysis, neural networks

## Abstract

Data processing in robotics is currently challenged by the effective building of multimodal and common representations. Tremendous volumes of raw data are available and their smart management is the core concept of multimodal learning in a new paradigm for data fusion. Although several techniques for building multimodal representations have been proven successful, they have not yet been analyzed and compared in a given production setting. This paper explored three of the most common techniques, (1) the late fusion, (2) the early fusion, and (3) the sketch, and compared them in classification tasks. Our paper explored different types of data (modalities) that could be gathered by sensors serving a wide range of sensor applications. Our experiments were conducted on Amazon Reviews, MovieLens25M, and Movie-Lens1M datasets. Their outcomes allowed us to confirm that the choice of fusion technique for building multimodal representation is crucial to obtain the highest possible model performance resulting from the proper modality combination. Consequently, we designed criteria for choosing this optimal data fusion technique.

## 1. Introduction

The emergence of our hyper-connected and hyper-digitalized world (IoT, ubiquitous sensing, etc.) requires any education organization to have the ability to handle a system that produces huge amounts of different data. A key area of research in multimodal data is the process of building multimodal representations, the quality of which determines the modeling and prediction of organizational learning.

Multimodal learning involves working with data that contain multiple modalities, such as text, images, audio, and numerical or behavioral data. The interest in multimodal learning began in the 1980s and has gained popularity since then. In one of the first papers on the subject [1], the authors demonstrated that acoustic and visual data could be successfully combined in a speech recognition sensor system. By integrating both of the modalities they outperformed the audio data model.

Further research has shown that modalities can be complementary and a carefully chosen data fusion leads to significant model performance improvements [2,3,4]. Furthermore, modalities might naturally coexist, and the analyzed machine learning (ML) problem cannot be solved unless all modalities are considered [5]. Finally, recent improvements in multisensor solutions [6] have offered high-quality modalities, which require an appropriate fusion technique to fit to the ML problem.

Choosing, or even designing, the right data fusion technique is fundamental to determining the quality and performance of data fusion strategies to be undertaken by data scientist teams in organizations. Moreover, data fusion strategies are the central concept of multimodal learning. This concept could be applicable to numerous machine learning tasks with all kinds of modalities (a so-called universal technique). According to [4], a universal approach has not been established yet. The research work conducted by the authors shows that all current state-of-the-art data fusion models suffer from two main issues. Either they are task-specific or too complicated or they lack interpretability and flexibility.

Our paper explores different types of data (modalities) that can be gathered by sensors serving a wide range of sensor applications. Data processing in robotics is currently challenged by addressing the urgent research problem: how can we effectively build multimodal and common representation in sensor systems? Consequently, this topic impacts the effectiveness of machine learning modeling for sensor networks.

We believe that the development of a universal technique requires analysis and many research experiments to understand the differences between the existing and most often used data fusion techniques. We claim that effective multimodal data fusion depends on the type of technique used to build this representation. We further develop decision criteria that define this dependency. We examine the most common techniques: late fusion, early fusion, and sketch. In the first technique—late fusion—all the modalities are learned independently and are combined right before the model makes a decision. The second technique—early fusion—combines all modalities and then learns the model. The last one—sketch—is similar to early fusion; however, modalities are transformed into common space instead of being concatenated. We perform the comparison in classification tasks. Finally, we define criteria and recommendations for choosing a multimodal data fusion technique. The chosen criteria comprise (1) the impact each modality has on the analyzed machine learning (ML) problem, (2) the type of the task, and (3) the amount of memory used while in the training and predicting phase. We conduct experiments on three datasets, Amazon Reviews [7], MovieLens25M, and MovieLens1M [8]. They encapsulate three modalities—textual, visual, and graph data—each has potential because each can bring unique information to the ML model.

In our paper, we contribute to the contemporary discussion on how to investigate the application of multimodal data fusion techniques in different production settings. We confirm that the choice of data fusion technique is crucial for maximizing the performance of the multimodal model. Consequently, we show conditions under which the combination of modalities outperforms any one of them used separately. Finally, we design decision criteria for choosing the optimal data fusion technique in classification tasks.

Thus, in the following Section 2 we state the problems of multimodality and present the employed datasets (Section 3). Finally, we experiment with the chosen multimodal fusion techniques (Section 4 and Section 5), provide conclusions in Section 6, and identify areas for future research in Section 7.

## 2. Related Work

### 2.1. Types of Modalities

This section introduces the types of modalities that can be encountered while working on any machine learning problem. According to [9], we identify four main groups of modalities:

- Tabular data: observations are stored as rows and their features as columns;
- Graphs: observations are vertices and their features are in the form of edges between individual vertices;
- Signals: observations are files of appropriate extension (images:.jpeg, audio:.wav, etc.) and their features are the numerical data provided within files;
- Sequences: observations are in the form of characters/words/documents, where the type of character/word corresponds to features.

Our study includes modalities coming from each of the identified groups: labels are an example of tabular data; reviews, titles, and descriptions represent textual data (sequences); images of products and movie posters are examples of visual data;, and relations between movies (movies seen by one user) are graphs. Table 1 introduces some other examples of works on various combinations of modalities. Additionally, in [10], the authors concentrated on the image segmentation task to learn the optimal joint representation from rich and complementary features of the same scene based on images retrieved from different sensors. All these studies showed no restrictions on modalities that can be studied together to solve a specific problem. Moreover, they proved that the combination of modalities boosts performance; the multimodal model achieved better results than the unimodal models.

Furthermore, these works encapsulate all multimodal fusion techniques that we examine in our research: early fusion, late fusion, and sketch. They are proven effective but have not been compared yet.

### 2.2. Multimodal Representation

Learning to represent is an unsupervised task, and there is no single way to describe a good representation. However, several works have identified the main features demanded while deriving any numerical representation of a given modality.

The problem of unimodal representation has already been solved with modality-dedicated models, such as BERT [17] for textual data, ResNet [18] for images, etc. However, a universal method that could be applied to any machine learning task when it comes to multimodal data has not been established [4].

Bengio et al. [19] characterized several features that an appropriate vector representation should possess, including:

- Smoothness: A transformation should preserve objects similarities, expressed mathematically as x≈y⇒f(x)≈f(y). For instance, the words “book” and “read” are expected to have similar embeddings;
- Manifolds: probability mass is concentrated within regions of lower dimensionality than the original space, e.g., we can expect the words “Poland”, “USA”, and “France” to have embeddings within a certain region, and the words “jump”, “ride”, and “run” in another distinct region;
- Natural clustering: categorical values could be assigned to observations within the same manifold, e.g., a region with the words “Poland”, “USA”, and “France” can be described as “countries”;
- Sparsity: given an observation, only a subset of its numerical representation features should be relevant. Otherwise, we end up with complicated embeddings whose highly correlated features may lead to numerous ambiguities.

For multimodal representation, ref. [20] identifies more factors that should be taken into account: (1) the similarity between individual modalities should be preserved in their joint representation and (2) robustness to the absence of some modalities; it should still be possible to create multimodal embedding.

### 2.3. Multimodal Data Fusion

Multimodal data fusion is an approach for combining single modalities to derive multimodal representation. A few issues should be taken into account [4] when it comes to fusing several modalities:

- Intermodality: the combination of several modalities, which leads to better and more robust model predictions [2];
- Cross-modality: this aspect assumes inseparable interactions between modalities. No reasonable conclusions can be drawn from data unless all their modalities are joined [21];
- Missing data: for some cases, particular modalities might not be available. An ideal multimodal data fusion algorithm is robust to missing modalities and uses others to compensate for the widespread information loss in recommender systems.

Classically, the existing multimodal representation techniques are divided into two categories [2,22]: early (feature) and late (decision) fusion. In the early feature approach, all modalities are combined. This is usually achieved by concatenating their vector representations at an initial stage, and then one model is trained [23]. In the case of late fusion, several independent models concerning each modality are trained, then their outputs are connected. The connection can be made arbitrarily. One can average the outputs and pick the most frequent one (in classification tasks), or concatenate them and build a model to obtain a final output [23]. Neither of these data fusion approaches can be described as the best one [22]; both have been proven to yield promising results in various scenarios.

#### 2.3.1. Deep Learning Models

The most popular multimodal fusion techniques are based on deep learning solutions. The authors of [4] describe such architecture ideas, along with their most representative cases. Four prominent approaches are deep belief nets, stacked autoencoders, convolution networks, and recurrent networks. However, despite their promising results in the field of multimodal data fusion, deep learning models suffer from two main issues [4]. Firstly, deep learning models contain enormous free weights, especially parameters associated with a modality that brings little information. This results in high resource requirements; an undesirable feature in a production scenario. Secondly, multimodal data usually come from very dynamic environments. Therefore, there is a need for a flexible model that can quickly adapt to all changes in data.

Enormous computational requirements and low flexibility suggest exploring other techniques applied to any task, despite the types of modalities. Furthermore, the authors of [4] suggest that these ideas can be combined with deep learning techniques and existing multimodal models to obtain state-of-the-art solutions, which would be applicable in every field and robust to all data imperfections (missing modalities, data distribution changes over time in a production case, etc.). According to [24], the best approach is based on deep learning; the challenge is modality fusion. One of the possible solutions is the use of hashing methods. The following section discusses the strengths and weaknesses of such algorithms.

#### 2.3.2. Hashing Ideas

Another promising approach in multimodal data fusion is associated with hashing models. They identify manifolds in the original space and then transform data to lower-dimensional spaces while preserving observation similarities. Such algorithms can construct multimodal representation on the fly and have been proven effective in information retrieval problems [3], recommendation systems [25], and object detection cases. The main advantages [3] of hashing methods are that they (1) are cost-effective in terms of memory usage, (2) detect and work within manifolds, (3) preserve semantic similarities between points, (4) are usually data-independent, and (5) are suitable for production cases as they are robust to any data changes.

Unfortunately, hashing methods struggle with one issue. The mapping of high dimensional data into much simpler representations can result in the loss of certain information about specific observations [3]. Therefore, it has to be verified if hashing ideas can be applied to other fields apart from similarity search tasks. Perhaps their ability to combine multiple modalities while maintaining low costs and robustness to data changes recompenses the lost information.

#### 2.3.3. Sketch Representation

The sketch representation has already been proven effective if fed with visual, behavioral, and textual data for the recommendation and similarity search tasks [25]. The idea of this representation comes from combining two algorithms: locality sensitive hashing and count-min sketch. All modalities are transformed into mutual space with the use of hash functions. Generally, a sketch is a one-hot sparse matrix containing all combined modalities. Hash functions make the representation modality independent, robust to missing modalities, and easily interpreted. Furthermore, modalities can be added to the sketch on the fly, which is extremely important in a production scenario.

**Figure 1:**
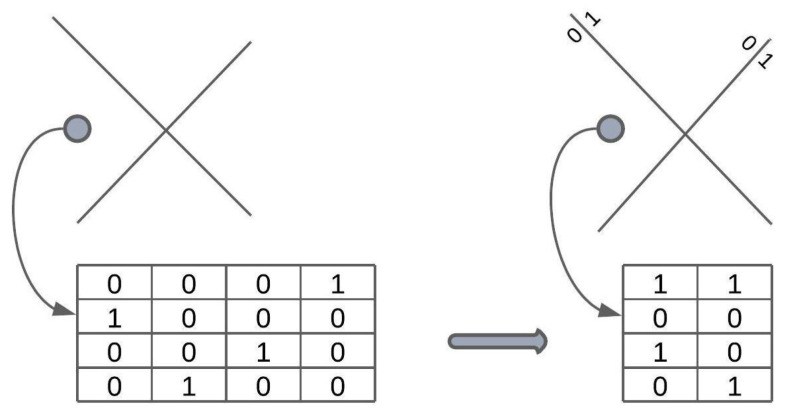
The idea of binarizing the sketch. Instead of representing an observation with a subspace ID, it can be represented as a set of binary features. Then, 0 and 1 represent where the point lies concerning a single hyperplane. Such a sketch consumes much less memory and perhaps preserves more information about a single observation.

In our research, we slightly modify this sketch representation to the binarized form, see Figure 1. Instead of representing an observation with a subspace ID, it can be represented as a set of binary features. Then, 0 and 1 represent where the point lies concerning a single hyperplane. Such a sketch should preserve more information about a single observation.

### 2.4. Multimodal Model Evaluation

Evaluating the multimodal data fusion algorithm is not straightforward, and no universal metric would measure the aspect of captured inter- and cross-modalities [21]. However, we can assess whether learning from multiple data types simultaneously enhances task performance.

The most popular way of verifying the quality of the multimodal fusion model [22] is to compare its performance scores (precision, AUC, etc.) to those achieved by models considering single modalities. With such an approach, we can state whether and to what extent combining modalities brings new information. We also aim to preserve all similarities between observations, i.e., similar observations should be comparable in their multimodal representations. Therefore, several works [23,25] have compared their multimodal models to NN algorithms, which serve as a good baseline. Lastly, multimodal models should be tested when adjusting additional modalities. In certain cases [23], adding new modalities slightly improves the results while the training time increases dramatically. As a result, the model might be unfeasible in production scenarios despite its excellent performance. Therefore, we should not only focus on the scores the model achieves but also consider its flexibility and simplicity. In our work, we compare three techniques for building multimodal representation in any sensor systems: late fusion, early fusion, and sketch. The main ideas of all of them have been described in this section. The next part introduces the datasets that were used for carrying out analyses.

## 3. Datasets

To evaluate different multimodal representations, we decided to compare them in classification problems, where we can quickly assess the amount of information each modality brought. Furthermore, we wanted to analyze production settings where the multimodality is expected to benefit the model, i.e., a model based on several modalities should obtain better performance than the unimodal model.

We found three datasets—Amazon Reviews, MovieLens25M, and MovieLens1M—applicable to our research design. They encapsulate three modality types in total: textual, visual, and graphs. Furthermore, in these datasets, each modality should be highly informative, e.g., it should be possible to classify a single product using any of its characteristics, i.e., the description, the title, or the image.

Table 2 presents the overall characteristics of the three used datasets (Amazon Reviews, MovieLens25M, and MovieLens1M) and the employed machine learning tasks. In our experiments, Amazon Reviews and Movielens25M were split at 0.6/0.2/0.2 into the training, validation, and test sets, respectively. With such a division, the results on test sets are representative (12,000 samples) while maintaining enough observations for training. The split of 0.8/0.2 (training and test sets, respectively) was used for MovieLens1M. Instead of one predefined validation set, MovieLens1M used the k-fold cross-validation (on the training set). We opted for cross-validation because this dataset was much smaller than the other two.

The **Amazon Reviews** dataset consists of products from Amazon. The data used in experiments are only a small subset of the original dataset (jmcauley.ucsd.edu/data/amazon/, accessed on 15 January 2021), i.e., the “Clothing, Shoes, and Jewellery” category. Namely, we randomly sampled 5000 products per each of the following categories: *Headdress, Boots, Jewellery, Wallet, Shirt, Dress, Underwear, Pants, Watch, Jacket, Sweater, and Luggage.* Intuitively, products within this category should be divergent enough. Furthermore, products within the fashion field can be expected to contain meaningful descriptions, titles, and, most importantly, images. Missing modalities characterize the dataset extracted from Amazon Reviews. Around 25% of descriptions are lost and 3% of titles are empty; however, all the records contain images.

The **MovieLens25M** dataset is the largest of the MovieLens family (https://grouplens.org/datasets/movielens/, accessed on 7 February 2021). It consists of 25 million ratings given to 62,000 movies by 162,000 users. Each movie is associated with genres and with one IMDb identifier. The metadata was collected based on such an identifier (the movie’s poster and plot and the visual and textual embedding, respectively). Some movies are subscribed to incorrect IMDb identifiers and were therefore removed. After preprocessing, the dataset in our experiment consisted of 60,763 unique movies. The existing modalities are movie poster (image), movie plot (text), and also views (graph).

There are some missing modalities: every movie has a plot, 63 movies lack a poster, and users in MovieLens25M did not rate 3231 movies. Missing modalities are proportional to class sizes, meaning the most considerable number of missing modalities is in the Drama class. Here, classes are highly imbalanced. Around 40% of all observations are dramas and 27% are comedies, whereas film-noir and IMAX movies are associated with less than 1% of observations. There is also a high number of movies that are not subscribed to any specific category, the *(no genres listed)* class.

**MovieLens1M** is a smaller version of the previously described dataset and consists of 6040 users that have rated approximately 4000 movies. However, in addition to user ratings, their demographic data are also available. This allows for investigating dependencies between watched content and user characteristics such as gender, age, or occupation. After preprocessing for our experiment, only two modalities are utilized: textual (the plot) and visual (the poster). Again, the data were downloaded based on IMDb identifiers associated with movies. There are no missing modalities. Classes are imbalanced; around 71.7% of users are males. This imbalance is considered during the evaluation using the MCC metric.

## 4. Research Design and Experiments

We performed three experiments with three classification tasks: multiclass, multilabel, and binary (see Table 2). The type of task was determined by the characteristics of the dataset prepared. The methods of carrying out the tests were described in each experiment (for textual embeddings, refer to: https://github.com/flairNLP/flair accessed on 15 January 2021).

### 4.1. Multiclass Classification—Amazon Reviews Dataset

Analyses on the Amazon Reviews Dataset consider the multiclass classification task based on two modalities—textual and visual. Classes are evenly distributed, and therefore the accuracy metric is used. The primary purpose of the experiments on this dataset is to compare multimodal fusion techniques based on neural networks:

- The late fusion, where modalities are treated independently. Here, models’ architectures are based on [26];
- Early fusion models, where embeddings of each modality are concatenated at the input level;
- The sketch, including the classical and binarized versions.

**Figure 2:**
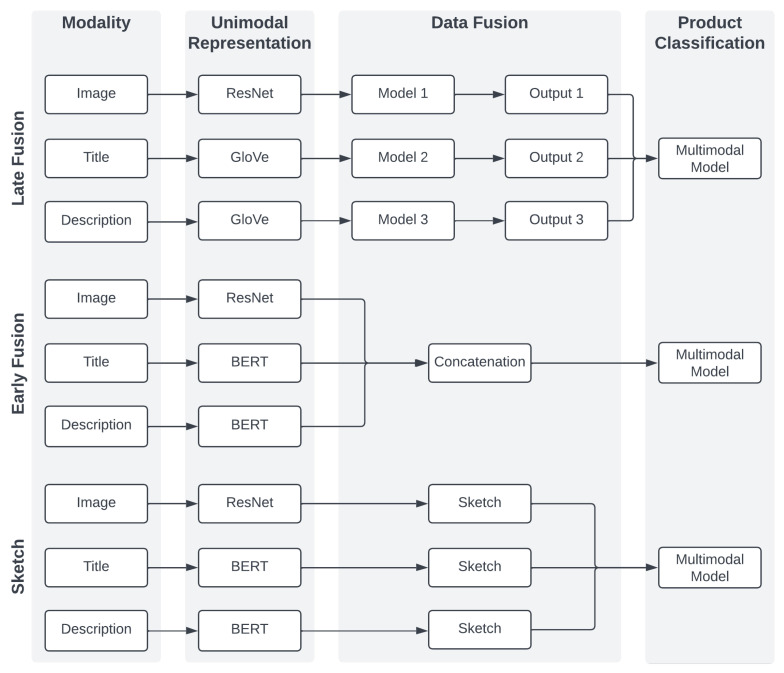
Here, we present the architectures used in the Amazon Reviews dataset. They are depicted in their trimodal forms. Bimodal and unimodal architectures look the same. The only difference is that unimodal models do not have a model on top of their outputs in the late fusion approach. Above, the output means the probabilities of observations belonging to respective classes.

Their overall architectures in our research design are visible in Figure 2. These approaches use Adam as the optimizer and a categorical cross-entropy loss function. The batch size was set to 32. All models were trained for ten epochs, except for the binarized sketch models, which were trained for 20 epochs. The learning rate differs per approach and equals $10^{-4},10^{-3},10^{-5}$, and $10^{-4}$ in the late fusion, the early fusion, the classical, and the binarized sketch approach, respectively.

#### 4.1.1. Late Fusion Models

While deploying ideas from [26], slight modifications are required from multilabel to multiclass classification tasks, i.e., a different loss function or number of used neurons within layers. Both the description and title are represented with GloVe. Image embeddings are created with ResNet50. All multimodal models are identical. The outputs from unimodal models were fed into a dense layer, with 20 units and ReLU as the activation function. Finally, a 12-unit dense layer for product classification was added. Therefore, the only difference between bimodal and trimodal models is the input size, which was equal to 24 and 32, respectively.

#### 4.1.2. Early Fusion

Here, each modality is firstly transformed into a numerical vector with BERT (*bert-base-uncased*) for both the titles and descriptions, resulting in 768 dimensional vectors. For image embeddings, ResNet50 was used. The last layer was removed, which results in 2048 dimensional vectors. In this approach, models have the same architecture, whether uni- or multi-modal. Therefore, the only difference is that in the multimodal case, appropriate embeddings are concatenated, resulting in input embedding sizes of 1536 (textual + textual), 2816 (textual + image), and 3584 (trimodal). The model architecture consists of three blocks: dense dropout layers, with 64 units; ReLU as the activation function; and the dropout layer, where the rate is set to 0.1. Subsequently, one dense layer is added with 64 units and ReLU. However, in this case, the dropout is not applied. Finally, there is the 12-unit dense layer with softmax, which is responsible for classification.

#### 4.1.3. Sketch Representation

Similar to the early fusion approach, BERT is used for the textual and ResNet50 for the visual data. Firstly, embeddings were transformed into sketches. The sketch depth was equal to 128 and the width was equal to 512. Then, sketches were flattened and concatenated in multimodal cases. The architecture is universal, despite the number of modalities. The model starts with two identical blocks consisting of a dense layer, a dropout layer, and batch normalization. The dense layer in the first block consists of 1024 neurons and the second consists of 512 neurons. After the second block, there is a 128-dimensional dense layer, followed by a dropout layer. These dense layers use ReLU, and the dropout rate is set to 0.2. Finally, a 12-dimensional dense layer with softmax is responsible for classification. The binarized version uses the same architecture to provide a fair comparison with the classical sketch.

### 4.2. Multilabel Classification—MovieLens25M

The Movielens25M dataset considers multilabel genre classification; its classes are highly imbalanced and correlated. Therefore, threshold-independent metrics are used for evaluation: the area under curve (AUC) and the mean average precision (mAP). Such an approach makes analyzed solutions independent from class threshold selection at testing time. Furthermore, to take the class imbalance into account, *micro* versions of these metrics are used: micro-AUC and micro-mAP.

**Figure 3:**
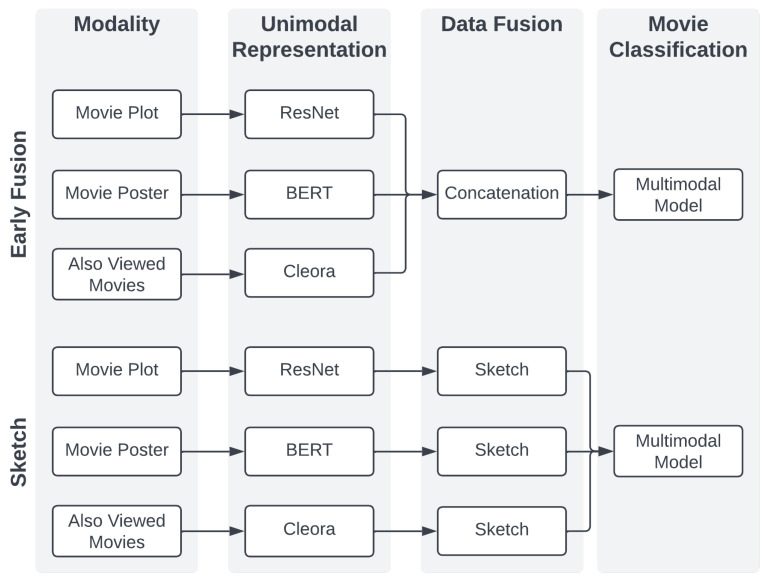
Architectures used in the MovieLens25M dataset.

The sketch and the early fusion representations were considered for a comparison. They both exploit the same architecture, which is described in the following section. Figure 3 illustrates both of the approaches.

Movielens25M encapsulates three modalities: graph, text, and images. The first one is encoded with Cleora. Cleora’s hyperparameters are set as follows: the embedding size was 1024 and the number of iterations was 1. According to [27], such parameters are optimal in preserving strong similarities between entities. Text data are represented with BERT (*bert-base-cased*) and visual data with ResNet50. Both sketches’ hyperparameters (depth and width) were set to 128.

The models consist of four dense layers. The first three use ReLU and have 1024, 512, and 128 neurons, respectively. The final, 20-dimensional layer is used for multilabel classification and therefore uses sigmoid. Models use the binary cross-entropy loss function the the Adam optimizer with a learning rate equal to $1^{-5}$, and its other parameters are set as default. Each model was trained for 20 epochs, apart from multimodal early fusion models and unimodal graph models, which were trained for 30 epochs. The batch size was set to 64. Every model was trained five times using the training and validation data and evaluated on the test data to obtain robust results.

### 4.3. Binary Classification—MovieLens1M

The task on MovieLens1M is a binary classification, predicting people’s gender based on their behavioral data (here, movies they had watched).

**Figure 4:**
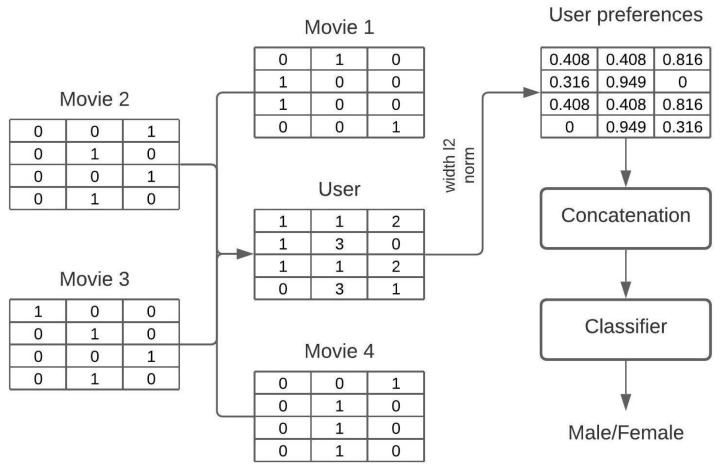
The architecture of gender classification. The user is represented as a sum of sketches. Then, a width-wise L2 normalization is performed. Finally, the sketch is flattened and fed into the classifier.

In the sketch representation, every movie is represented with the sketch and the user as a sum of such sketches, see Figure 4. However, unlike the recommender scenario, the output is a number between 0 and 1, representing the probability that the user is a male or a female.

Every user has rated (watched) at least 20 movies. Movies are represented with the plot and the poster, as in MovieLens25M. Then, users are represented by the content they watch. Once a user sketch is created, it is flattened and fed into logistic regression with the regularization parameter *C* set to 0.1. The sketch width was equal to 512, while the depth was adjusted to the modality type, equal to 210 for the movie plot and 128 for the movie poster. In the bimodal model, the flattened sketches are concatenated. As a result of the class imbalance, the Matthew correlation coefficient (MCC) is used for evaluation. Movie plots are embedded with BERT (*bert-base-cased*) and posters are embedded with ResNet50.

## 5. Results

Table 3, Table 4 and Table 5 shows our results for different tasks and datasets. We can see that only in one dataset—Amazon Reviews—do all the used modalities improve the final results. The other tests on MovieLens datasets show that posters reduce the results, and intuitively we can imagine that the posters do not have much substantial information about movies.

**Figure 5:**
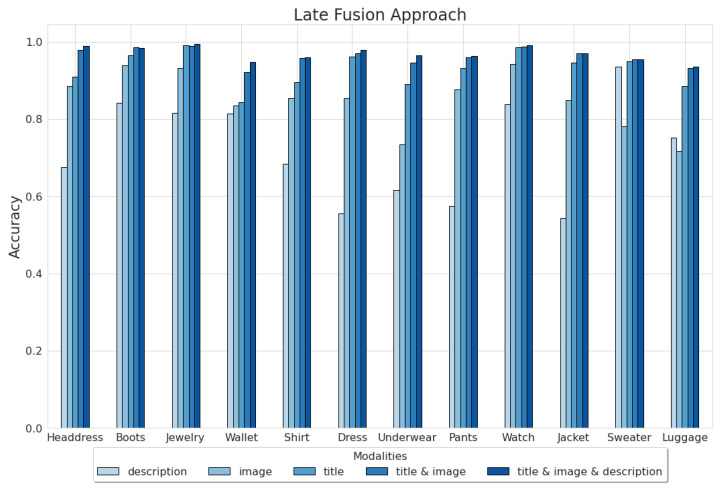
The late fusion models results concerning categories.

Since each modality’s impact on the model performance is not so clear in the Amazon Reviews dataset, we decided to explore them thoroughly concerning each category, see Figure 5. The analyzed results were obtained by the late fusion approach, which achieved the best results.

The bimodal and trimodal models outperform unimodal models in every category (accuracy metric). The most significant benefit from exploiting multiple modalities can be noticed within classes, where unimodal models yield close results, for instance, the *Wallet* class. On the other hand, categories, where one of the unimodal approaches is undeniably better, do not gain much from multimodality, e.g., the *Watch* class.

Surprisingly, despite the absence of around 25% of the data concerning the *Sweater* category, the description model significantly outperforms the image model within this class. This example shows that each modality, even ones with missing data, can yield unique information and be helpful until proven otherwise.

In most cases, the trimodal model does not significantly outperform the bimodal based on title and image. Furthermore, trimodal model training takes twice the time than that of the bimodal model. In the case of this thesis, this is not relevant, since it concerns training models on 48,000 observations. However, in real-world scenarios, the trimodal model could be too expensive for the slight improvement it provides. Hence, only titles and images can be considered valuable in this task.

In the case of gender classification, the highest MCC (0.543) indicates that the proposed architecture might be successfully used to solve demographic classification problems. It could be a powerful tool, for instance, for e-commerce or user-generated platforms. By modeling user characteristics, companies could personalize their offers to maximize their profits. On the other hand, users will benefit from personalized content. However, more experiments on larger datasets should be performed to confirm the usefulness in the production scenario.

To the best of the authors’ knowledge, our work classifies users based on the content of their interests for the first time (see experiment on MovieLens1M for gender classification). To date, the existing approaches [28] have considered only the behavioral data, which in the case of MovieLens1M is present as ratings that users give to movies. Our experiment shows that it is possible to model user characteristics with the content in which the users are interested. Though only simple classifiers were tested, it was proven that our approach is reasonable.

All the tests show that combining several modalities boosts model performance; however, the data must be meaningful for the given task. If possible, additional modalities are suggested, as they may provide new insight into the analyzed data. Our research concludes that multiple data modalities should be utilized cautiously, as some might yield little information (here, movie posters are redundant for genre and gender classification).

The main conclusions from the tests are summarized in Table 6 and explained in the following. These conclusions are made upon the following criteria: each modality’s influence on the analyzed machine learning (ML) problem, the type of the ML task, and the memory constraints during the training and predicting phase.

Tests on the Amazon Reviews dataset prove that late fusion in nearly all scenarios is the best. Here, the reason is probably the type of textual data. Many descriptions and titles contain words that are sufficient on their own for a good prediction. Therefore, in situations where one of the modalities is dominant, we believe that the best approach would be late fusion. The model efficiently exploits the information from the most informative modality and can expand it using additional modalities.

On the other hand, the early fusion approach can reveal information from the combination of the modalities (interaction between modalities), which can be interdependent. Therefore, in scenarios where several modalities affect the model equally, their interactions might reveal hidden information and hence should be learned.

Our experiments showed that the sketch performs worse in typical classification problems. However, this representation can be the least consuming in storing data representations. Furthermore, when the number of used modalities increased, the performances of sketch-based models almost always increased. Possibly, this improvement could be maintained until reaching state-of-the-art performance. If such behavior was confirmed, the sketch representation could offer ground-breaking perspectives in the world of Big Data with highly accurate models and optimized storage consumption.

## 6. Conclusions

This paper explored three of the most common techniques for building multimodal data representations, (1) the late fusion, (2) the early fusion, and (3) the sketch, and compared them in classification tasks. Our paper explored different types of data (modalities) that could be gathered by sensors serving a wide range of applications. Our experiments were conducted on Amazon Reviews, MovieLens25M, and MovieLens1M datasets. The results showed that a data fusion strategy is crucial for maximizing multimodal model efficiency. In the case of the Amazon Reviews dataset, the late fusion approach substantially outperformed the other systems by approximately 3%.

Results on all datasets confirmed that multimodality could outperform any unimodal representation. The performance of models improved from 0.919 to 0.969 in accuracy on the Amazon Reviews dataset and from 0.907 to 0.918 in AUC on the MovieLens25M dataset. However, our experiments on both MovieLens datasets indicated the importance of meaningful input data to the given task. Analyses showed that movie posters did not contain much substantial information about movies.

To the best of our knowledge, we are the first to propose three unique selection criteria for choosing a technique appropriate to the ML task (see Table 6), which should be taken into account while building multimodal representation. These criteria are the impact each modality has on the task, the type of the task, and the amount of used memory. Furthermore, using these criteria, we summarized the three explored techniques, offering recommendations that can be used by all scientists working on any multimodal task.

Our work did not entirely exploit the problem of choosing the proper technique for building multimodal representations. However, we confirmed that such a choice matters, and we designed recommendation criteria that could support it. In the next section, we present the directions of future research which, in combination with our work, could set the basis for developing a universal multimodal fusion technique.

## 7. Future Research Directions

Given the recent improvements in sensor systems, more high-quality modalities are available. This creates both opportunities and challenges that should be addressed to develop a universal multimodal data fusion technique. We highlighted three major areas of research: (1) designing a multimodal benchmark, (2) extending multimodal applications, and (3) increasing multimodal interpretability.

**Designing a multimodal benchmark,** similar to GLUE [29] for NLP applications, is crucial for establishing the standards of multimodal representation research. It would confirm all conclusions made so far in previous and future works on this subject. Multibench [30] could be considered a promising candidate for such a benchmark. It is a collection of fifteen datasets which span across seven different applications. Extending Multibench with new applications and establishing state-of-the-art models along with a dynamic leaderboard could be the next step.

**Identifying new multimodal applications** is essential to create a universal data fusion technique. For instance, recent research on biomedical applications [31,32] exposed the limitations and strengths of existing multimodal fusion strategies. Consequently, exploring other applications would highlight the work that still needs to be carried out in the field of building multimodal representation.

**Multimodal interpretability** is the process of understanding multimodal deep neural nets [33,34]. Despite their outstanding performance, the complexity, opaqueness, and black-box nature of the deep neural nets limit their social acceptance and usability. Hence, understanding the impact of individual modalities, and especially their interactions, is fundamental for any multimodal application.

## Figures and Tables

**Table 1:** Examples of multimodal tasks.

Modalities	Article	Overview
Images, time series, and tabular	[11]	Prediction of Alzheimer’s disease based on magnetic resonance imaging and positron emission tomography (images) that are performed multiple times on one patient within specified periods of time (time series). Patient demographics and genetic data are also taken into account (tabular).
Audio, video, and event streams	[12]	Behavioral analysis and emotion and stress prediction. Analyzed data consist of 45-min recordings of students during the final exam period. They are recorded with the use of cameras (video), thermal physiological measurements of the heart, breathing rates (event streams), and lapel microphones (audio).
Text and images	[13]	Question answering based on images containing some textual data.
Images, text, and graphs	[14,15,16]	Outfit/movie recommender systems. Movies are recommended based on plot (text), poster (image), liked and disliked movies, and cast (graphs). Outfits are chosen based on product features in images and text descriptions.

**Table 2:** Dataset characteristics.

Dataset Name	Size	Modalities	Task
Amazon Reviews	60,000	- Textual (product description and title) - Visual (product image)	Product classification (multiclass)
MovieLens 25M	60,763	- Textual (movie plot) - Visual (movie poster) - Graph (movies seen by one user)	Genre classification (multilabel)
MovieLens 1M	6040	- Textual (movie plot) - Visual (movie poster)	Gender classification (binary)

**Table 3:** Amazon Reviews dataset—results.

Modality	Late Fusion	Early Fusion	Sketch	Sketch Binarized
Title	0.919±0.002	0.857±0.002	0.78±0.002	0.822±0.004
Description	0.706±0.003	0.662±0.001	0.613±0.003	0.636±0.004
Image	0.836±0.005	0.851±0.002	0.77±0.002	0.812±0.003
Title and description	0.962±0.001	0.91±0.002	0.851±0.002	0.868±0.006
Image and description	0.927±0.001	0.898±0.003	0.847±0.002	0.859±0.003
Image and title	0.962±0.001	0.93±0.002	0.885±0.002	0.903±0.004
Image, title, and description	**0.969±0.001**	**0.94±0.002**	**0.912±0.002**	**0.914±0.002**

Amazon Reviews dataset—the accuracy of each approach for each modality combination. Results are averaged over ten runs.

**Table 4:** MovieLens25M—results.

	Early Fusion Approach		Sketch Approach	
**Modality**	**AUC**	**mAP**	**AUC**	**mAP**
Text	0.907 ± 0.001	0.549 ± 0.001	0.877 ± 0.003	0.465 ± 0.003
Image	0.815 ± 0.002	0.350 ± 0.001	0.778 ± 0.003	0.297 ± 0.003
Graph	0.824 ± 0.001	0.382 ± 0.002	0.797 ± 0.003	0.323 ± 0.003
Text and graph	**0.918 ± 0.001**	**0.592 ± 0.002**	0.883 ± 0.002	0.484 ± 0.002
Text, graph, and image	0.915 ± 0.001	0.583 ± 0.002	0.880 ± 0.002	0.482 ± 0.003

Results of the early fusion and the sketch on MovieLens25M. Results are averaged over five runs.

**Table 5:** MovieLens1M—gender classification results.

Modality	Matthew Corellation
Movie poster	0.518
Movie plot	**0.543**
Movie plot and poster	0.532

Logistic regression results of the gender classification on test set.

**Table 6:** Comparing technique selection criteria for building multimodal representations.

Technique	Notes and Recommendations:
Late fusion	- Use when one modality is dominant (its unimodal model gives significantly better results than others); - Use when every unimodal model achieves high performance; - Preferred in classification tasks (probabilities from unimodal models can be easily combined), such as in [26].
Early fusion	- Use when modalities are dependent, i.e., no reasonable conclusions can be made unless modalities are studied together (e.g., questions to pictures [13]); - Use when all unimodal models yield similar results; - Easy to use pre-trained models; the model input is their early fusion.
Sketch	- Memory efficient (sketches can be very short vectors, stored and used efficiently, and have low memory usage); - Suggested for information filtering problems, such as recommender systems [25]; - Easy to use pre-trained models as inputs to sketching technique.

The recommendations are constructed concerning three criteria: the influence that each modality has on the analyzed machine learning (ML) problem; the type of the ML task; and the memory constraints during the training and predicting phase.

## Data Availability

The data presented in this study are openly available: Amazon Reviews—https://jmcauley.ucsd.edu/data/amazon/, DOI: 10.1145/2872427.2883037, accessed on 15 January 2021 MovieLens1M, MovieLens25M—https://grouplens.org/datasets/movielens/, DOI: 10.1145/2827872, accessed on 7 February 2021.

## Abbreviations

The following abbreviations are used in this manuscript:

MDPI	Multidisciplinary digital publishing institute
ML	Machine learning
AUC	Area under curve
NN	Nearest neighbour
mAP	Mean average precision
MCC	Matthew correlation coefficient
